# The species of *Thalerosphyrus* Eaton, 1881 (Insecta, Ephemeroptera, Heptageniidae, Ecdyonurinae) in Java and Sumatra, with some comments on the diversity of the genus in the Oriental Realm

**DOI:** 10.3897/zookeys.420.7904

**Published:** 2014-06-25

**Authors:** Michel Sartori

**Affiliations:** 1 Zoologisches Museum und Biozentrum Grindel, Martin-Luther-King-Platz 3, D-20146 Hamburg, Germany; 2 Museum of Zoology, Palais de Rumine, Place Riponne 6, CH-1005 Lausanne, Switzerland

**Keywords:** *Thalerosphyrus determinatus*, *Thalerosphyrus sinuosus*, *Thalerosphyrus lamuriensis*, *Ecdyonurus sumatranus*, distribution, Bali, Sumbawa, nymph, eggs, SEM

## Abstract

Three species belonging to the genus *Thalerosphyrus* Eaton, 1881 are reported from Java and Sumatra. The nymphs of *Th. determinatus* (Walker, 1853) from Java, *Th. sinuosus* (Navás, 1933) from Java and Sumatra and *Th. lamuriensis* Sartori, 2014 from Sumatra are redescribed. The egg morphology of the three species is also presented for the first time. A key to the nymphs is proposed. General considerations on the composition of the genus *Thalerosphyrus* in the Oriental Realm are given. The distribution of the genus is greatly expended, and currently ranges over the Himalaya and Sumbawa in the Sunda Islands.

## Introduction

The genus *Thalerosphyrus* was created by Eaton (1881) to accommodate the species *Baetis ? determinata* Walker, 1853 described on the basis of a single male imago from Java. Later on (Eaton 1885), the same author added also *Baetis ? torrida* Walker, 1853 known by a single female imago from the Philippines. *Thalerosphyrus determinatus* was recorded later by Ulmer (1913) also from Java, and redescribed in detail, with abdominal patterns, coloration and drawing of the genitalia. Another species was described from Java by Navás under the name *Ecdyonurus ? sinuosus* Navás, 1933 on the basis of a single female imago, and then transferred to the genus *Thalerosphyrus* by Ulmer (1939) who described the male imago and reported the species also from Sumatra. In the same work (Ulmer 1939), the author described *Thalerosphyrus determinatus* and *Thalerosphyrus sinuosus* in the nymphal stage. Dang (1967) created the genus *Ecdyonuroides* for a peculiar nymph he collected in Vietnam (*Ecdyonuroides vietnamensis*) which possesses extremely well developed posterolateral expansions on the abdomen. He recognized the similarity with a nymph described by Ulmer (1939) under the name *Ecdyonurus sumatranus* and designated Ulmer's species at the type species of his new genus. Later on, Braasch and Soldán (1984) put *Ecdyonuroides* in synonymy with *Thalerosphyrus* on the basis of a rearing of *Ecdyonuroides vietnamensis* nymph which gave a male imago with *Thalerophyrus* characters and proposed the new combination *Thalerosphyrus vietnamensis*. In the following years, two other *Thalerosphyrus* species were described: *Thalerosphyrus bishopi* (Braasch & Soldán, 1986b) from West Malaysia, and *Thalerosphyrus flowersi* (Venkataraman & Sivaramakrishnan, 1987) from southern India, both at the adult and nymphal stages.

Braasch and Soldán (1986a) described a new genus (*Asionurus*) from Vietnam and showed that the nymph described by Ulmer (1939) under the name *Thalerosphyrus sinuosus* was incorrectly associated with adults of this species and that the nymph actually belonged to this new genus and therefore proposed to call this taxon *Asionurus ulmeri*. Wang and McCafferty (2004) suggested that the nymph described by Ulmer (1939) as *Thalerosphyrus determinatus* was wrongly associated and should be the nymph of *Thalerosphyrus sinuosus* according to abdominal patterns.

The concept of the genus *Thalerosphyrus* is far from being clear, because the type material of the type species, *Thalerosphyrus determinatus* is in bad state, missing all legs but one as well as the abdomen (hence the genitalia) (Kimmins 1960). The uncertainties about the actual status of the genus *Thalerosphyrus* let Kluge (2004) to consider it as *incertae sedis*, referring only to the nymphs of *Thalerosphyrus sumatranus*, *Thalerosphyrus vietnamensis* and *Thalerosphyrus flowersi* as belonging to his non ranking taxon Ecdyonuroides/g(1) characterized by their developed posterolateral expansions of the abdomen.

When revising Ulmer's collection in the Zoologisches Museum in Hamburg, Sartori (2014d) restudied the type material of *Ecdyonurus sumatranus* Ulmer, 1939 and showed that the holotype belonged to the genus *Rhithrogena* Eaton, 1881, and thus put *Ecdyonuroides* Dang, 1967 in synonymy with *Rhithrogena* and proposed a new name for the nymph as *Thalerosphyrus lamuriensis*.

Within the ongoing revision of Ulmer's collection (Sartori 2014a; b; c; d), we have reinvestigated all material of *Thalerosphyrus* deposited in the Museum of Zoology in Hamburg. Despite the above mentioned uncertainties, we follow the *Thalerosphyrus* concept proposed by Ulmer (1913; 1924; 1939) because his redescription of *Thalerosphyrus determinatus* is in accordance with Eaton's (1885) diagnosis, especially body and wing lengths and hind leg ratios. The nymphs of the species found in Java and Sumatra are described based on this historical material as well as on specimens recently collected.

## Material and methods

Original material studied here is deposited in the following institutions:

ZMH Zoologisches Museum und Biozentrum Grindel, Hamburg, Germany

MZL Musée cantonal de zoologie, Lausanne, Switzerland

LIPI Lembaga Ilmu Pengetahuan Indonesia (Indonesian Institute of Sciences), Museum of Zoology, Bogor, Indonesia.

In the absence of adequate life stages to link nymph and adults as previously proposed by Sartori (2014a; c; d), eggs were extracted from either female imago or subimago for *Thalerosphyrus determinatus* and *Thalerosphyrus sinuosus* because no mature female nymphs were available, and from a mature female nymph for *Thalerosphyrus lamuriensis* as no alate stage of this species are known for sure.

Ontogenetic stage association relies thus on the following assumptions; three nymphal forms present together with three different egg morphologies, one species found only on Java, one on Java and Sumatra and the latter only on Sumatra.

Drawings were made with the help of a camera lucida taken from stereomicroscope Leica DM 750 and pictures from microscope Zeiss Axioscop 2 or Visionary Digital Passport II. Final digital drawings were performed on Adobe Illustrator CS6. For scanning electronic microscope (SEM) pictures, the eggs were dehydrated, carbon coated, and observed under a LEO 1525 at 5.00 kV; maxillae were dehydrated, critical point dried, and then platinum coated, and observed under a FEI Quanta 250 at 5.00 kV. Final plates were assembled in Adobe Photoshop CS6.

## Results

### Thalerosphyrus

Taxon classificationAnimaliaEphemeropteraHeptageniidae

Eaton, 1881

#### Nymphal diagnosis.

Medium to large Heptageniidae (up to 20 mm) with contrasting color patterns.

Head broad and thickened anteriorly (Figs 2, 5, 8); labrum (Figs 16–17) small, wider than long, without conspicuous median incision; mandibles (Figs 18–19) with outer margin covered with numerous thin setae, outer and inner incisors subequal in length, outer one saw-like on both sides, inner one trifid, left mandible with tuft of setae above mola; maxillae with 3-segmented palp, ventral surface of galea-lacinia covered with numerous long setae (Fig. 25), which appear entire in optical microscope, but are slightly feathered in SEM, crown of the galea-lacinia with 20–25 comb-shape setae, median ones bearing 12–17 teeth (Fig. 26), distal dentisetae bifid and fimbriate, as the proximal one (Figs 23–24); hypopharynx with robust lingua and well developed superlinguae bend backwards (Figs 27–28); labium with rhomboid glossae (Figs 20–22), paraglossae regularly curved, apex not bend backwards and moderately expended laterally.

**Figures 1–3.**
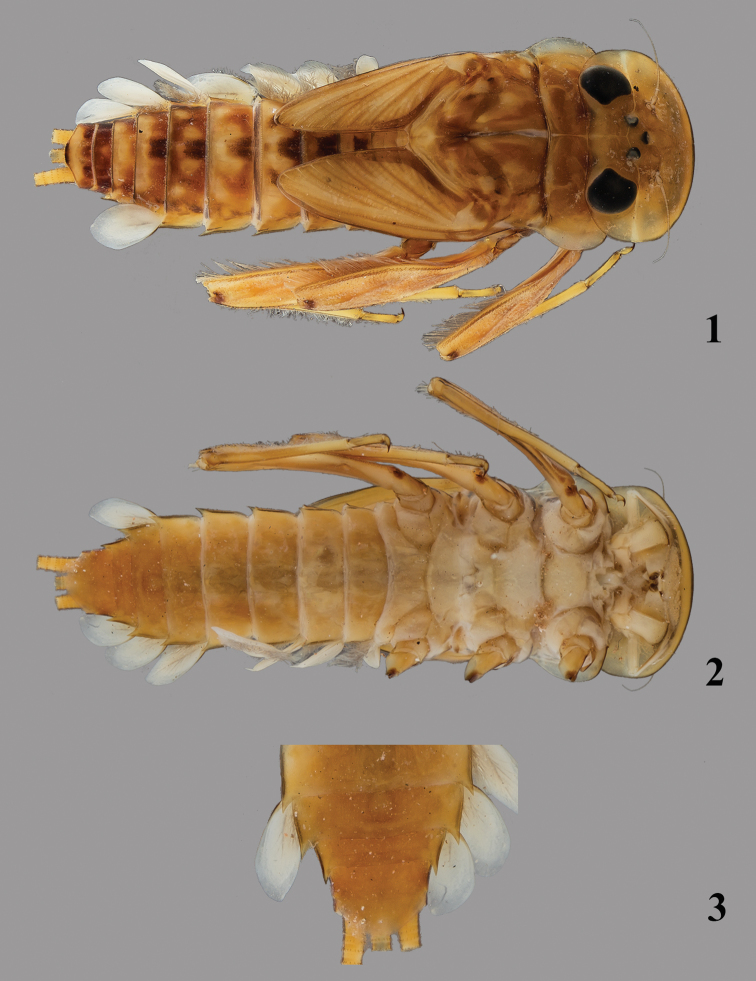
*Thalerosphyrus determinatus* (Walker, 1853). **1** Habitus in dorsal view **2** Habitus in ventral view **3** Detail of abdominal segments VI–IX in ventral view.

**Figures 4–6.**
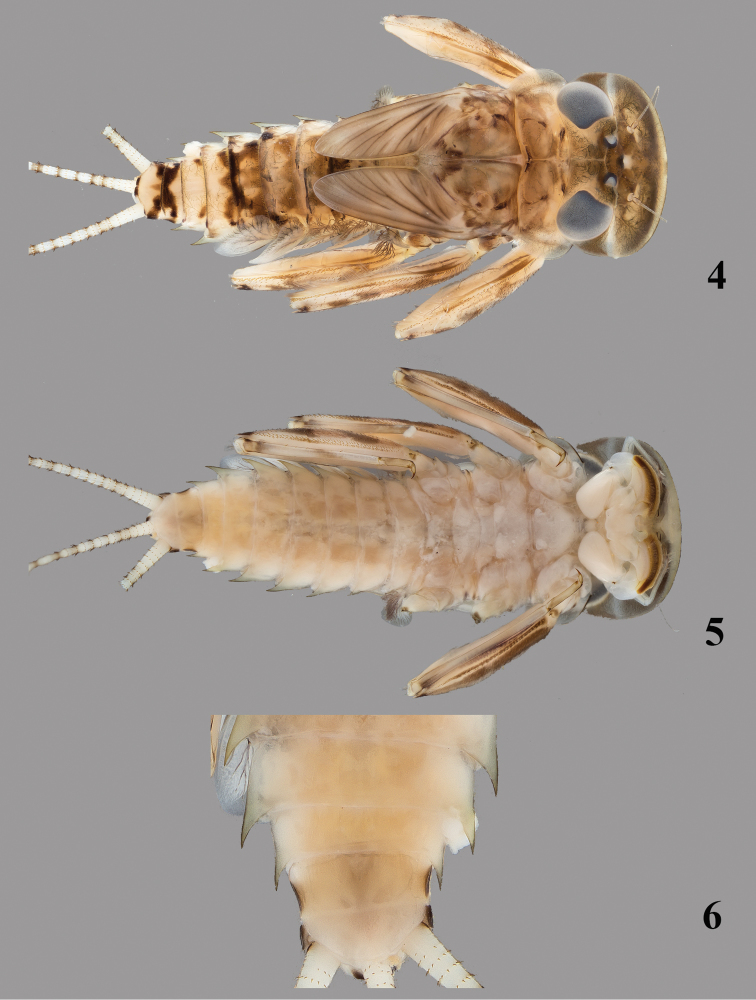
*Thalerosphyrus sinuosus* (Navás, 1933). **4** Habitus in dorsal view **5** Habitus in ventral view **6** Detail of abdominal segments VI–IX in ventral view.

**Figures 7–9.**
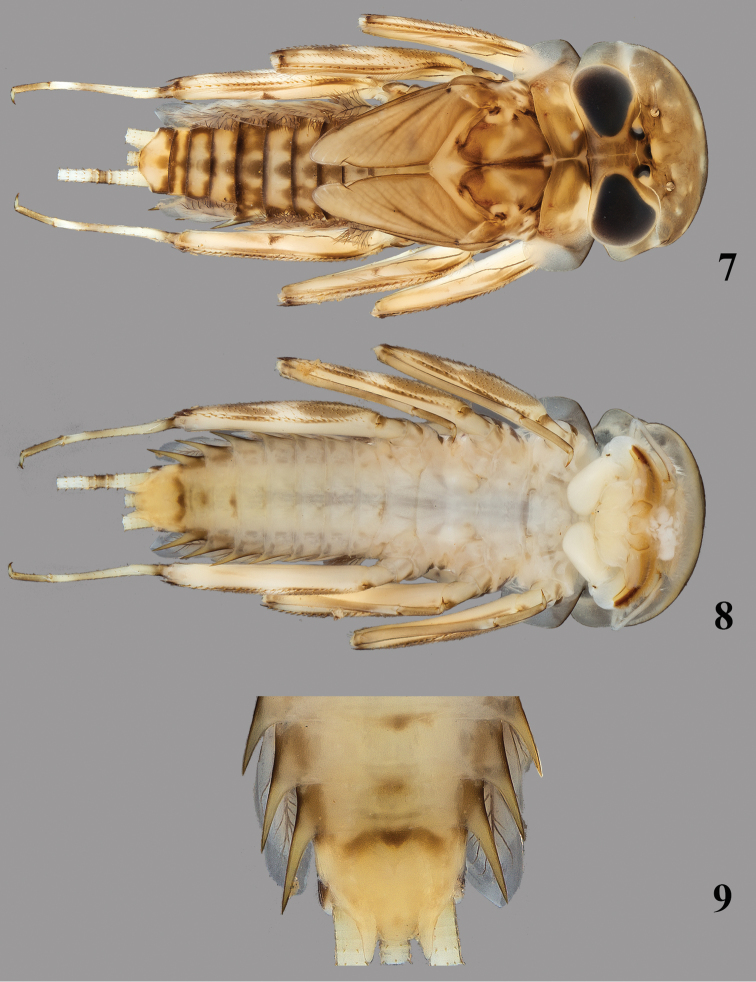
*Thalerosphyrus lamuriensis* Sartori, 2014. **7** Habitus in dorsal view **8** Habitus in ventral view **9** Detail of abdominal segments VI–IX in ventral view.

Thorax with pronotum slightly to greatly enlarged laterally; supracoxal spurs acute and well developed especially on mid- and hindlegs; femora rather similar between the three pairs of legs, row of stout and pointed bristles on inner and outer margins, no thin setae present; outer margin of fore tibia with few thin setae on proximal fourth, mid tibia with a row of thin setae on outer margin almost to tarsi, hind tibia (Figs 30, 32, 34) with two rows of thin setae, one on the outer margin, one in submarginal position, spine-like bristles absent or present.

Abdomen with posterolateral projection generally greatly enlarged from segment III to VII or VIII (Figs 3, 6, 9); posterior margin of tergites (Figs 35–37) with large and pointed teeth, microdenticles present and generally numerous; all gills asymmetrical (Figs 38–49), gills I–VI with plate-like and extremely developed fibrillose parts, gill VII only plate-like; terminal filament well developed, cerci whitish with more or less enlarged brown bands; segments with whorls of stout and pointed setae.

#### Discussion.

The scattered setae on the ventral surface of the maxilla indicate clearly that *Thalerosphyrus* belong to the subfamily Ecdyonurinae. The presence of acute supracoxal spurs, the anterior margin of the head thickened and generally well developed posterolateral projections of the abdomen are discriminating characters according to Webb and McCafferty (2008). To these we can add the shape of the gills II–V (VI) strongly asymmetrical and wider than long, with fibrillose part well developed. In the Oriental Realm, *Thalerosphyrus* could be confused with *Compsoneuriella* Ulmer, 1939 because of the acute supracoxal spurs, but is easily told by the much more developed posterolateral projections of the abdomen, the higher number of comb-like setae on the crown of the galea-lacinia, the shape of the gills which are never so wide in *Compsoneuriella*, and by the shape of the distal dentisetae, which are simple and not fimbriate in *Compsoneuriella* (Sartori 2014c).

#### Species included.

*Thalerosphyrus determinatus* (Walker, 1853): Java

*Thalerosphyrus sinuosus* (Navás, 1933): Java, Sumatra

*Thalerosphyrus vietnamensis* (Dang, 1967): Vietnam

*Thalerosphyrus bishopi* Braasch & Soldán, 1986: West-Malaysia

*Thalerosphyrus flowersi* Venkataraman & Sivamarakrishnan, 1987: South India

*Thalerosphyrus lamuriensis* Sartori, 2014: Sumatra

The species described by Ulmer (1926) as *Thalerosphyrus melli* from China has been recently assigned to another genus as *Epeorus melli* (Ulmer) by Zhou et al. (2007); the species *Thalerosphyrus torridus* (Walker, 1853) described based on a single female imago from the Philippines most probably belong to the genus *Afronurus* (Braasch 2011); the species *Thalerosphyrus separatus* Nguyen & Bae, 2004 and *Thalerosphyrus ethiopicus* Soldán, 1977 described from Vietnam and Sudan respectively, have been suggested to be also members of the genus *Afronurus* by Webb et al. (2006).

#### Distribution.

The genus *Thalerosphyrus* is endemic to the Oriental Realm. It is known from India, through Southeast Asia (Thailand, Vietnam, West Malaysia), up to Sumbawa in the Sunda Islands (see below), suggesting, as for *Rhithrogena* (Sartori 2014d), that the Wallace line is not a barrier to the dispersal of some Ephemeroptera. The genus is however not currently reported from Sulawesi (Edmunds and Polhemus 1990). According to Braasch (2011), *Thalerosphyrus* is also not recorded from the Philippines, and its presence on the island of Borneo is only based on few data and no named species are known (Braasch 2011); in the MZL collections is a single nymph (Sabah, Mesilau River, 8 km north of Kundessan, 2100 m, 1.VIII.1985, J.T. & D.A. Polhemus leg) which is clearly related to *Thalerosphyrus lamuriensis*, but complementary material is needed before any definitive answer can be found. In the MZL collections is also deposited a single nymph from Nepal (Nawakot & Sindhu Districts, Patibhanjyang Village, elev. ca 6000’, 10.IX.1968, C. Wiens leg) which expands the distribution of the genus to the Himalaya.

### Thalerosphyrus determinatus

Taxon classificationAnimaliaEphemeropteraHeptageniidae

(Walker, 1853)

Thalerosphyrus determinatus Ulmer, 1939, (nymph, pro parte)

#### Material examined.

2 nymphs, Java, Diengplateau, stream Seraju (D13), ca 1950 m a.s.l., 5.VI.1929, Prof. Thienemann leg. [ZMH]; 1 nymph entirely mounted on microscopic slide, Java, Gedeh Panggerango, Tjisarua, 1050 m, 10.VIII.1930, Dr. Lieftinck leg [ZMH]; 1nymph, Java, Java Barat Province, rocky stream at Cibodas (CL 2186), 1300 m, 3.XI.1985, J.T. & D.A. Polhemus leg [MZL]; 1 nymph, Bali, Baturiti, Desa Antapan, 815 m, 8°19.34'S, 115°11.61'E, 9.X.2009 (BLI005), M. Balke & D. Amran leg [MZL]; 1 nymph, Sumbawa, Nusa Tenggara Barat Province, Madsewu River, 2 km above Badindi, 61 km NW of Bima (CL 2174), 750 m, 20.X.1985, J.T. & D.A. Polhemus leg [MZL].

Eggs extracted from a female imago (caught together with a male imago) and identified by Ulmer as *Thalerosphyrus determinatus*: West Java, Tjibodas, Tjiwalen Bridge, 1400 m, 4.IX.1932, Dr Lieftinck leg [ZMH].

#### Description of the nymph.

Body size: up to at least 14.5 mm (not full grown nymph).

Coloration pattern: see Figs 1–2.

*Head.* Labrum moderately expended laterally, less than 4 times larger than long, with rounded apexes (as in Fig. 16); dorsal surface and anterior margin covered with long and thin setae; ventral surface with a median arch of less than 10 strong and pointed setae. Crown of the galea-lacinia of the maxillae composed of ca. 25 comb-shape setae, the median ones bearing 12–15 teeth. Right mandible with 5–6 bifid and fimbriate setae below the inner incisor and ca. 10 long simple and thin setae below the mola; left mandible with 8–9 simple and fimbriate setae below the inner incisor and ca. 9–10 long simple and thin setae below the mola. Hypopharynx with robust lingua bearing a tuft of small setae, superlinguae densely covered with long and thin setae replaced before the apex by very small setae up to the lower part of the superlinguae (Fig. 27). Labium with glossae rhomboid, slightly concave on their outer margin near apex (Fig. 20), dorsal surface with three stout setae and numerous thin and simple setae.

*Thorax.* Pronotum weakly expended laterally (Fig. 1). Femora with submarginal rows of pointed bristles on the inner and outer margins, increasing in numbers from the fore to the hind leg. Bristles on the upper face of hind femora arrow-shaped, clearly pointed (Fig. 29). Hind tibia (Fig. 30) without any bristles in outer marginal or submarginal position. Tarsal claw with 2–3 teeth.

*Abdomen.* Posterolateral expansions not developed on segments I–II, increasing in size from segment III to VII where it may reach the middle of segment VIII, shorter on segment VIII (Fig. 3) and comparable proportionally to those of segments V–VI. Gill I (Fig. 38) with elongated and rounded plate, ca 2.5× longer than wide; gill IV strongly asymmetrical (Fig. 39), wider than long, gill VI and VII oval and asymmetrical with obtuse apex (Figs 40–41). Posterior margin of tergites with irregular pointed teeth, and numerous microdenticles (Fig. 35). Cerci rather unicolor medium brown, some segments darker in the proximal half.

#### Description of the eggs.

**Figures 10–15.**
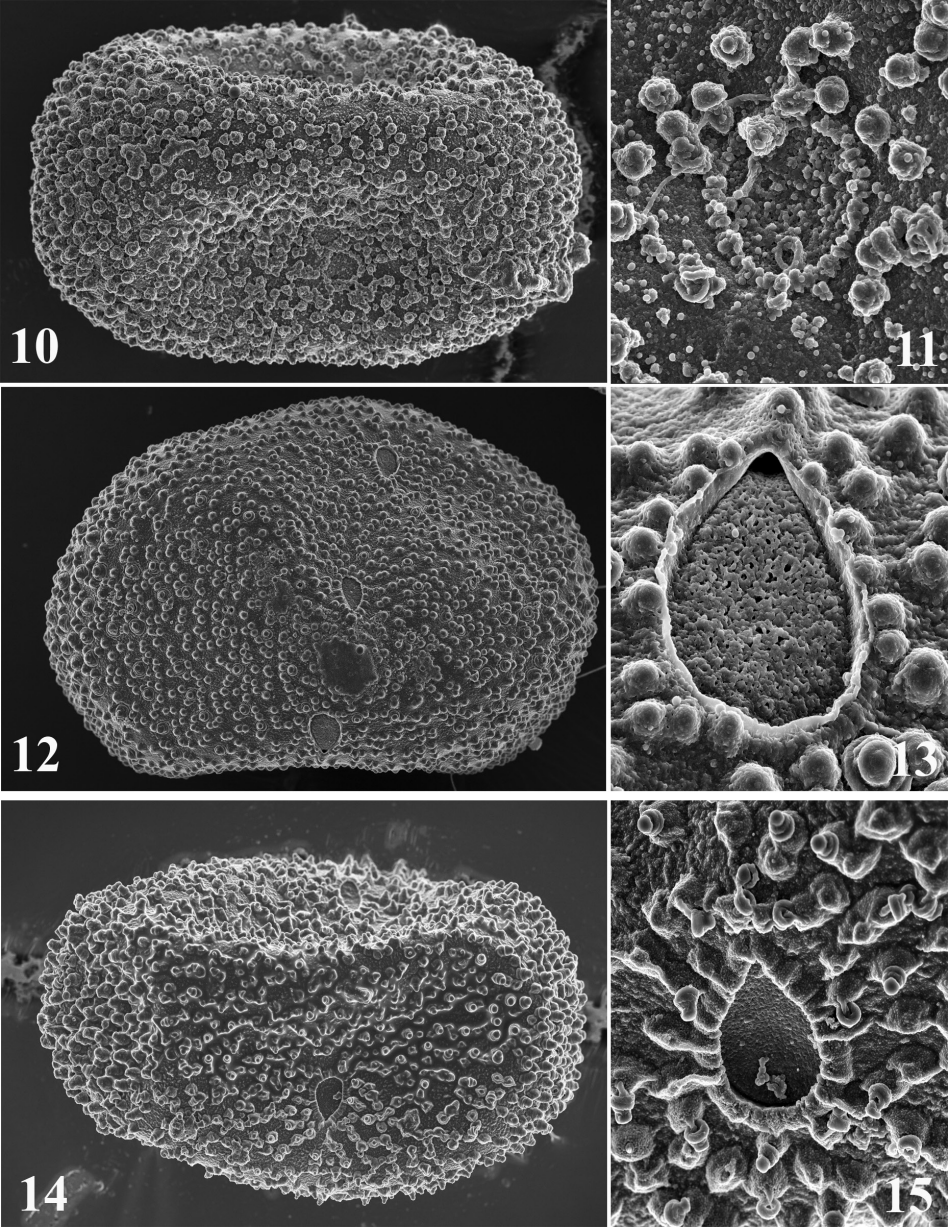
Egg structure of *Thalerosphyrus determinatus* **(10–11)**, *Thalerosphyrus sinuosus* **(12–13)**, *Thalerosphyrus lamuriensis* **(14–15). 10, 12, 14** Egg in toto **11, 13, 15** Details of the micropyle and chorionic structures.

Size: ca 120 µm × 75 µm; chorion regularly covered by small KCT'S, (1.0–1.5 µm), a little bit larger at poles (Fig. 10), and by microgranules (< 0.3 µm); margin of micropyle irregular and formed by microgranules (Fig. 11).

#### Discussion.

The abdominal pattern of the nymph is the one which is the closest to the one of the male imago redescribed by Ulmer (1924). According to Wang and McCafferty (2004) and our own observations (see below), the illustration of the abdominal patterns of the nymph (Ulmer 1939, fig. 402) as *Thalerosphyrus determinatus* does not belong to this species, nor do any of Ulmer's, other drawings.

The species may be easily recognized from its relatives mainly by the weak posterolateral expansions of the abdomen, and the absence of bristles on the outer margin of the hind tibiae.

*Thalerosphyrus determinatus* as defined here is the less common species found in the investigated area. However it is reported from Bali and Sumbawa for the first time. The species is absent from Sumatra and seems to live in middle to high altitudes, based on the few available data.

### Thalerosphyrus sinuosus

Taxon classificationAnimaliaEphemeropteraHeptageniidae

(Navás, 1933)

Thalerosphyrus determinatus Ulmer, 1939, (nymph, pro parte)

#### Material examined.

4 nymphs, two partially mounted on two microscopic slides, Sumatra, Singkarak, stream at Subanpass (F20), 1000 m, 4.III.1929, Prof. Thienemann leg [ZMH]; 1 nymph, Sumatra, Tjurup, Kali Dzernih, forested stream (M9), 7.V.1929, Prof. Thienemann leg [ZMH]; 1 nymph, Sumatra, Ranau, stream in primary forest (R25c), 29.I.1929, Prof. Thienemann leg [ZMH]; 2 nymphs, one partially mounted on a microscopic slide, Java, Gurung Ungaran, XII. 1909, Jacobson leg [ZMH]; 1 nymph, Java, Kali Tjiwalen near Tjibodas, 1350 m, in mosses and dead leaves (FY7f), 10.VII.1929, Prof. Feuerborn leg [ZMH]; 1 nymph, West Java, stream in Tjibodas, under the “mountain garden” (FY14c), 15.VII.1929, Prof. Feuerborn leg [ZMH]. [All specimens *sub. nom* *Thalerosphyrus determinatus* det. Ulmer].

10 nymphs, Java Tengah, Wonosobo-Kertek village road, creek, 800 m, 7°21.68'S, 109°55.67'E, 10.X.2011 (JVA011), M. Balke leg. [LIPI, MZL]; 2 nymphs, Sumatra Barat, Sijunjung / Muara area, forest, 488 m, 00°40.10'S, 101°07.26'E, 10.XI.2011 (UN7), M. Balke leg [MZL]; 7 nymphs, one entirely mounted on a microscopic slide, Sumatra Barat, Universitas Andalas campus, forest stream, 360 m, 00°54.67'S, 100°28.38'E, 8.XI.2011 (UN1), M. Balke leg [MZL]; 2 nymphs, Sumatra Barat, Lubukbargalung, Lubuk Paraku River, 50 km south Solok, 420 m, 100°32.50'E, 0°56.75'S, 25.V.2010 (SU5), J.-M. Elouard leg [MZL].

Eggs extracted from a female imago: Java, Buitenzorg, 13.II.1932, Dr Lieftinck leg [ZMH], and from a female subimago: Western Sumatra, Danau di Atas, stream near the road, 1000–1100 m (FF20e), 16.III.1929, Prof. Feuerborn leg [ZMH] and identified by Ulmer as *Thalerosphyrus sinuosus*.

#### Sequence data.

One specimen from Sumatra (SU5) and one from Java (JVA011) have been used for the study by Vuataz et al. (2013) under the name “*Thalerosphyrus*” in figures and “*Thalerosphyrus* sp.” in table S1, with voucher numbers “340SuTh” and “346JaTh” respectively, with one or two mitochondrial (CO1, 16S) and two to four nuclear genes (28S, H3, wg, EF-1α) sequenced. Access numbers in GenBank are:

**Table:** 

Voucher #	CO1	16S	28S	H3	wg	EF-1α
340 SuTh	HE651394	HE651430	HE651453	HE651512	HE651485	HE651535
346JaTh	HF536601			HF536587	HF536594	

#### Description of the nymph.

Body size: up to at least 10.5 mm (not full grown nymph).

Coloration pattern: see Figs 4–5.

**Figures 16–22.**
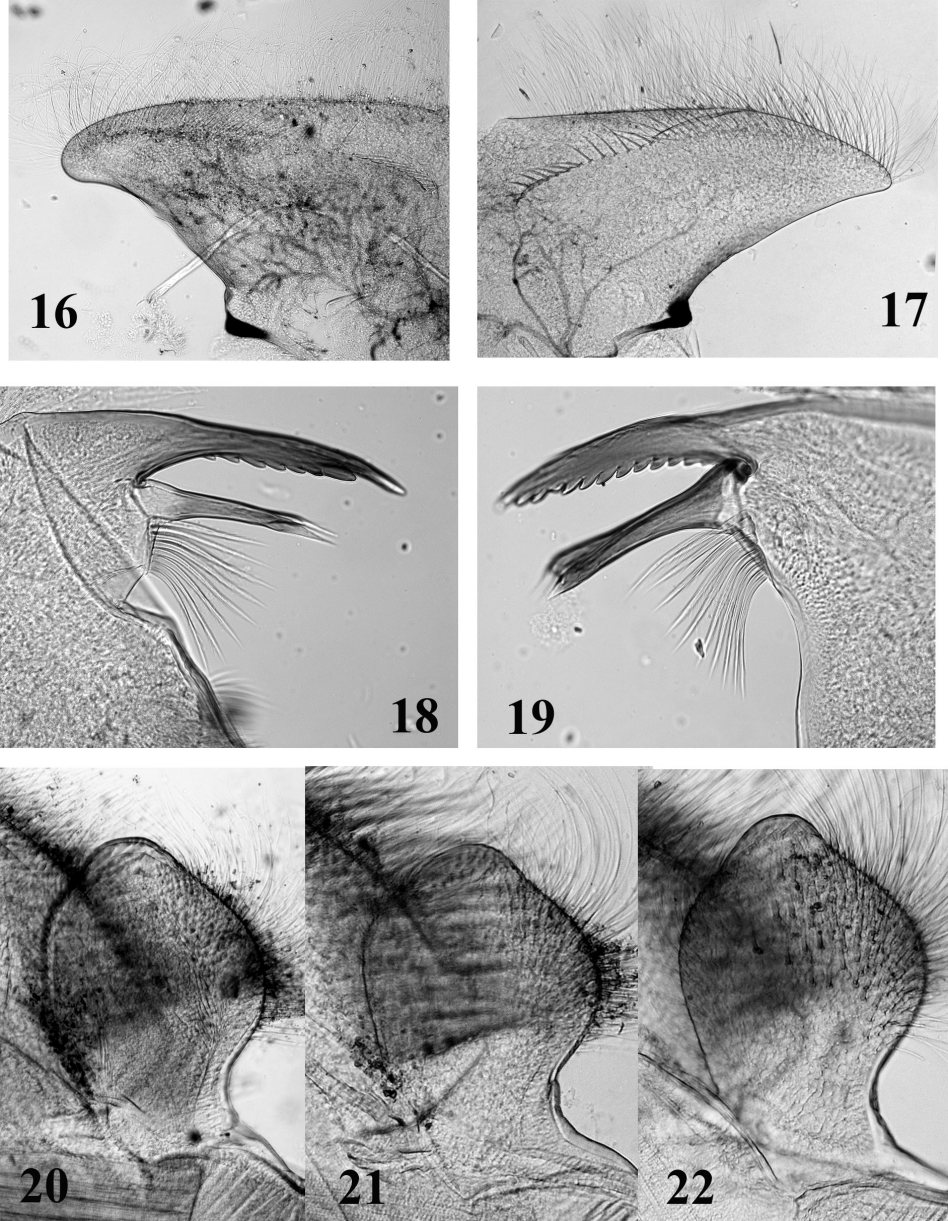
Mouthparts structure of *Thalerosphyrus determinatus* **(20)**, *Thalerosphyrus sinuosus* **(16, 21)** and *Thalerosphyrus lamuriensis* **(17, 18, 19, 22). 16–17** Hemi-labrum **18** Left mandible **19** Right mandible **20–22** Labial glossa.

*Head.* Labrum slightly expended laterally, ca 3.5 times larger than long, with rounded apexes (Fig. 16); dorsal surface and anterior margin covered with long and thin setae; ventral surface with a median arch of ca 10 strong and pointed setae. Crown of the galea-lacinia of the maxillae composed of ca 25 comb-shape setae, the median ones bearing 12–15 teeth (Fig. 26). Right mandible with 7–8 fimbriate setae below the inner incisor and ca. 5–6 long simple and thin setae below the mola; left mandible with 10–11 simple and fimbriate setae below the inner incisor and ca. 8–9 long simple and thin setae below the mola. Hypopharynx with robust lingua bearing a tuft of small setae, superlinguae densely covered with long and thin setae replaced before the apex by very small setae up to the lower part of the superlinguae. Labium with glossae rhomboid, slightly concave on their inner margin near apex (Fig. 21), dorsal surface with three stout setae and numerous thin and simple setae.

**Figures 23–26.**
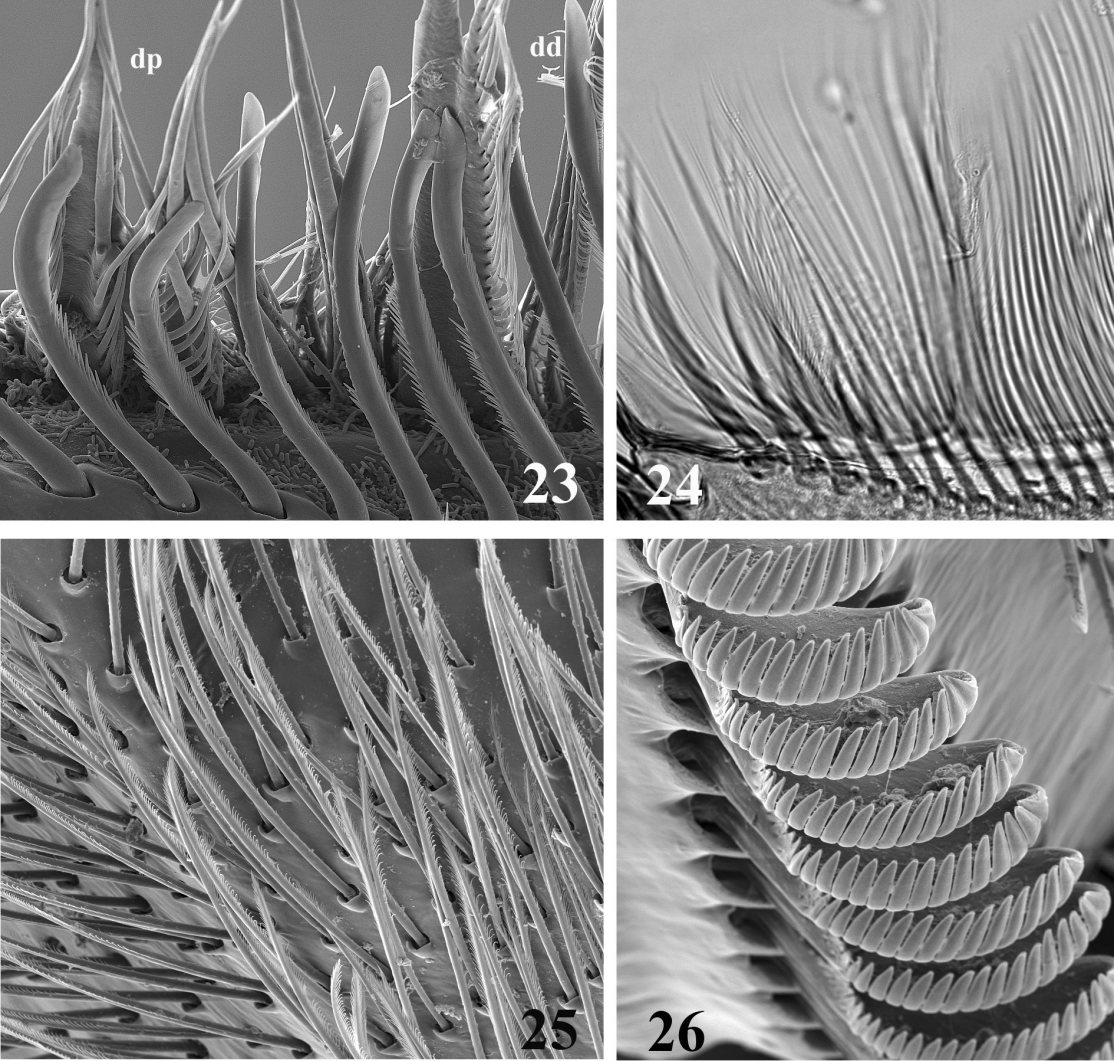
SEM **(23, 25, 26)** and optic **(24)** pictures of maxillar structure. **23–24** Dentisetae of *Thalerosphyrus lamuriensis* dp: proximal dentisetae, dd distal dentisetae **25** Scattered setae on the ventral face of the galea-lacinia of *Thalerosphyrus sinuosus* **26** Comb-shape setae on the crown of the galea-lacinia of *Thalerosphyrus sinuosus*.

*Thorax.* Pronotum slightly expended laterally and posteriorly (Fig. 4). Femora with submarginal rows of pointed bristles on the inner and outer margins, increasing in numbers from the fore to the hind leg. Bristles on the upper face of hind femora arrow-shaped, clearly pointed (Fig. 31). Hind tibia with a row of 6–7 arrow-shaped bristles in submarginal position (Fig. 32). Tarsal claw with 2–3 teeth.

**Figures 27–34.**
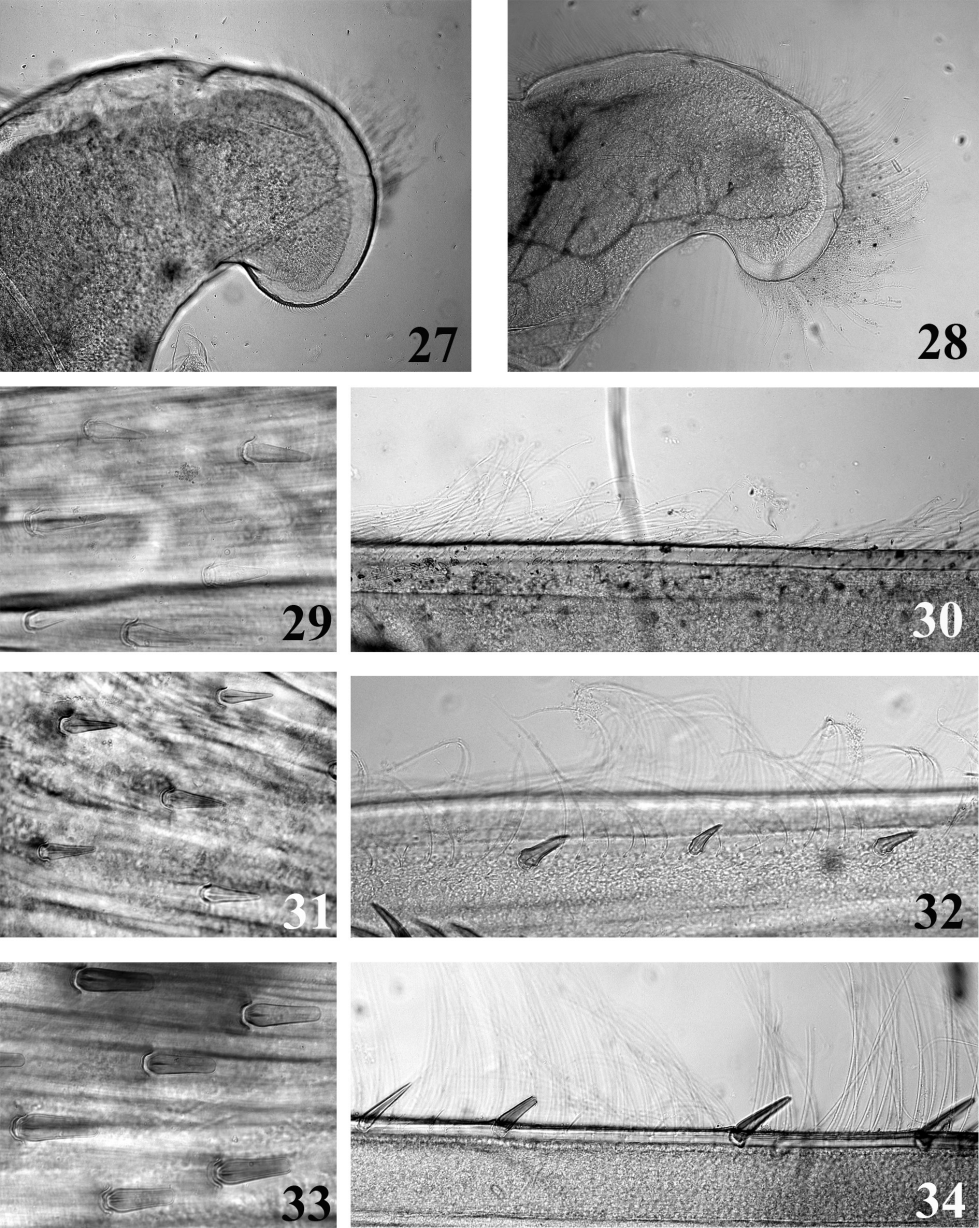
Mouthpart **(27–28)** and thoracic **(29–34)** structures of *Thalerosphyrus determinatus* **(27, 29, 30)**, *Thalerosphyrus sinuosus* **(31, 32)** and *Thalerosphyrus lamuriensis* **(28, 33, 34). 27–28** Apex of superlingua of hypopharynx **29, 31, 33** Bristles on the dorsal face of hind femur **30, 32, 34** Outer margin of hind tibia.

*Abdomen.* Posterolateral expansions not developed on segment I, weakly developed on segment II, strongly developed on segment III and increasing in size up to VII where they may be as long as segment VIII, shorter on segment VIII and smaller proportionally to those of segments III (Fig. 6). Gill I with elongated and rounded plate, less than two times longer than wide (Fig. 42); gill III–VI strongly asymmetrical, wider than long (Figs 43–44), gill VII oval and asymmetrical with inner concave margin near apex (Fig. 45). Posterior margin of tergites with irregular pointed teeth, and numerous microdenticles (Fig. 36). Cerci whitish in proximal part, with dark brown segment every two or three, distal part more uniformly medium brown.

**Figures 35–37.**
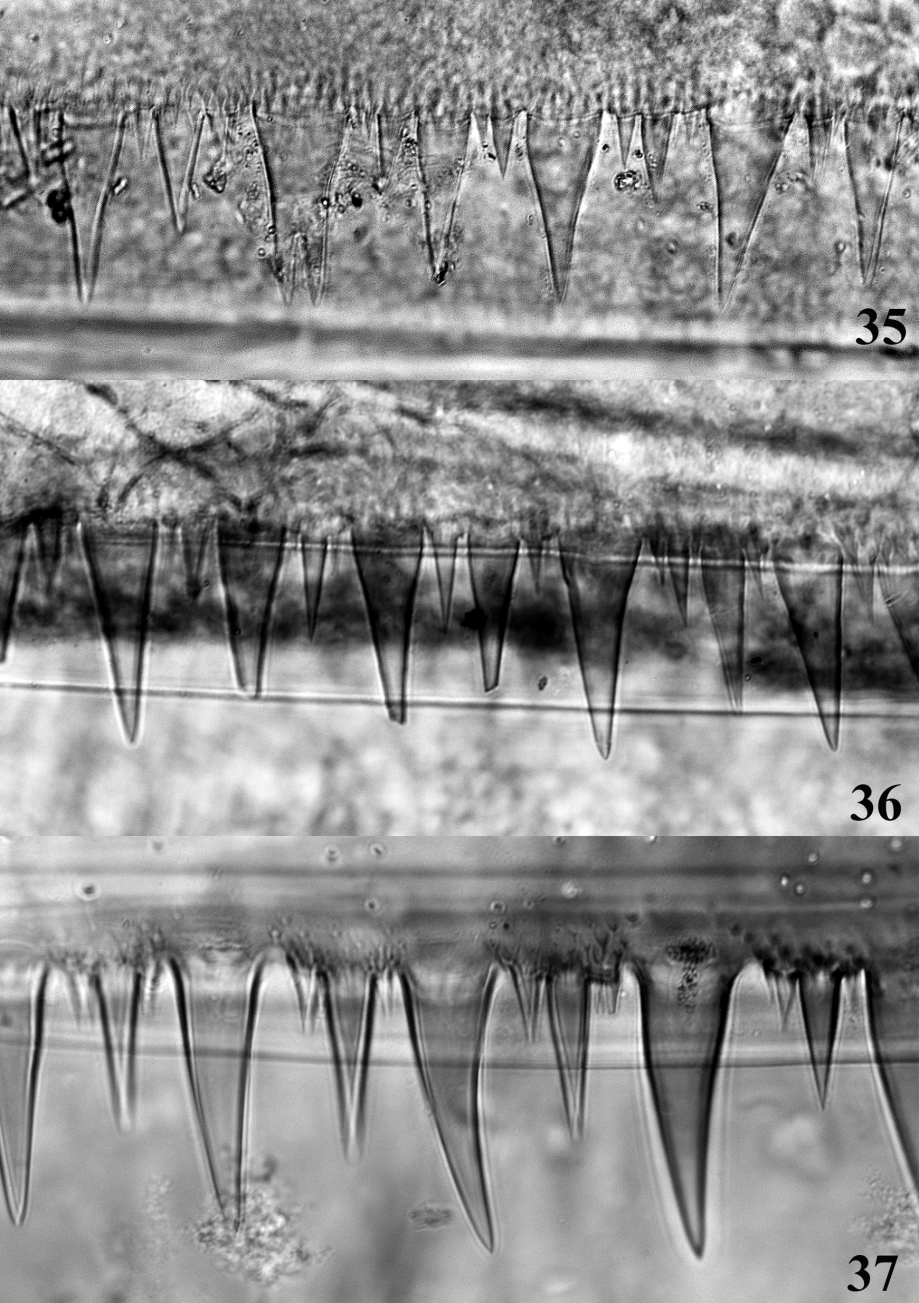
Posterior margin of abdominal tergite IV. **35** *Thalerosphyrus determinatus* **36** *Thalerosphyrus sinuosus* **37** *Thalerosphyrus lamuriensis*.

**Figures 38–49.**
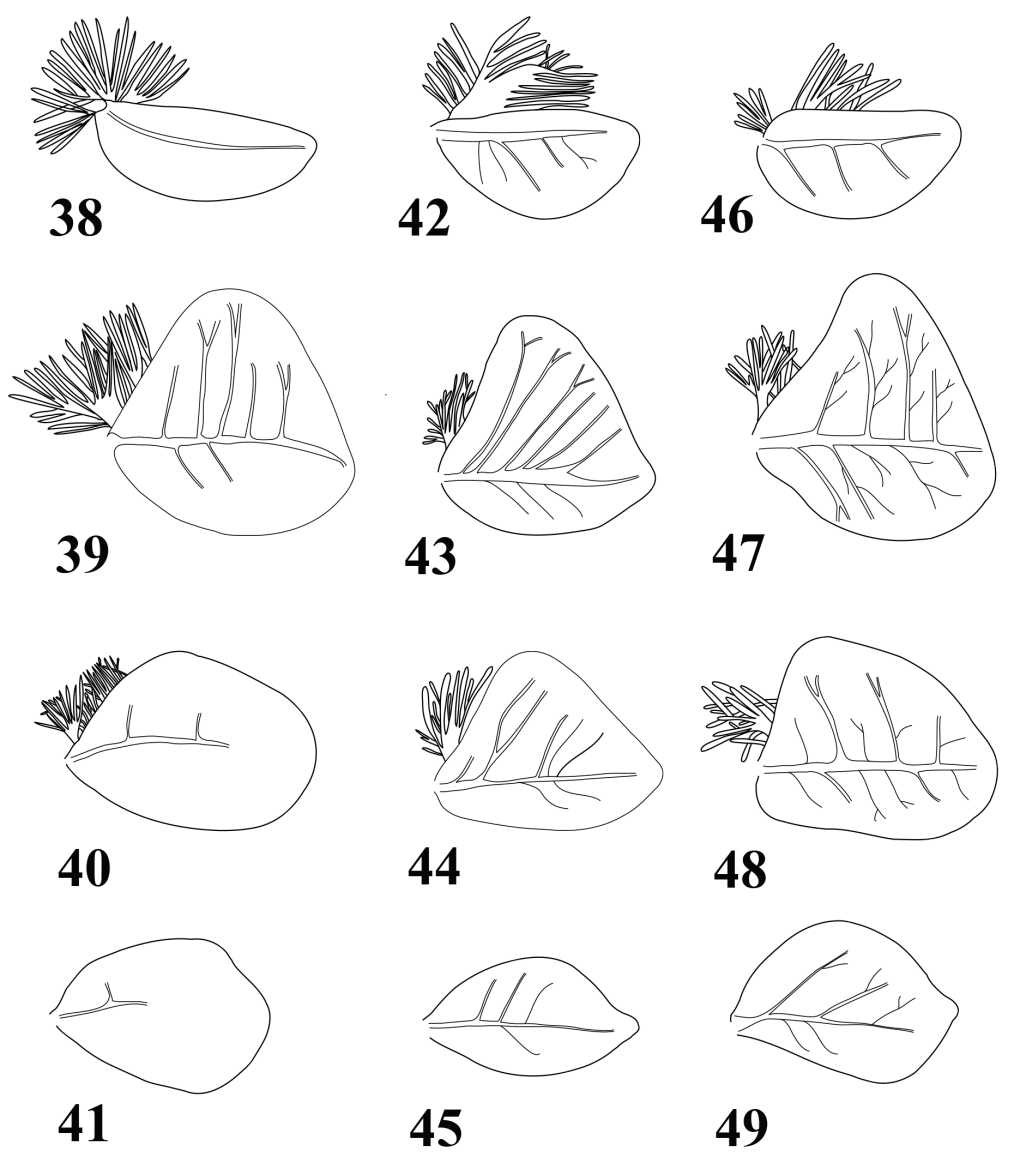
Gills of *Thalerosphyrus determinatus* **(38–41)**, *Thalerosphyrus sinuosus* **(42–45)** and *Thalerosphyrus lamuriensis* **(46–49). 38, 42, 46** Gill I **39, 43, 47** Gill IV **40, 44, 48** Gill VI **41, 45, 49** Gill VII.

#### Description of the eggs.

Size: ca 130–140 µm × 85–90 µm; chorion regularly covered by small KCT'S, (1.5–2.0 µm), a little bit larger at poles (Fig. 12), and by mesogranules (1.0 µm); margin of micropyle smooth and entire (Fig. 13).

#### Discussion.

The nymph mentioned here includes what Ulmer (1939) described as the nymph of *Thalerosphyrus determinatus*; the material is composed of three slides made by Ulmer himself and most of the drawings (Ulmer 1939, figs 403–418) were based on them. It appears that Ulmer confused the two species, and this is also because he made no slide preparation of the true *Thalerosphyrus determinatus*. *Thalerosphyrus sinuosus* as defined here is closely related to *Thalerosphyrus determinatus*, but can be easily separated by the shape of the posterolateral expansions of the abdomen, the shape of the gills, the shape of the glossae, and by the presence of arrow-shaped bristles on the hind tibiae.

The eggs of *Thalerosphyrus sinuosus* differ from those of *Thalerosphyrus determinatus* by the margin of the micropyle and by the presence of mesogranules on the chorion.

*Thalerosphyrus sinuosus* is present on Java and Sumatra. We cannot confirm the occurrence of the species outside these two islands, although based on egg morphology, and some partial details of the nymph (Boonsoong and Braasch 2013), the species could be present in Thailand, but supplementary description of the nymph is needed.

### Thalerosphyrus lamuriensis

Taxon classificationAnimaliaEphemeropteraHeptageniidae

Sartori, 2014

Ecdyonurus sumatranus Ulmer, 1939, (nymph, not female adult)
Thalerosphyrus determinatus Ulmer, 1939, (nymph, pro parte)
Thalerosphyrus sumatranus Braasch & Soldan, 1984 (nymph)

#### Material examined.

Besides the type material mentioned in Sartori (2014d), the following specimens have been examined.

1 nymph, Sumatra, Singkarak, stream at Subanpass (F19), 1000 m, 4.III.1929, Prof. Thienemann leg [ZMH]; 3 nymph, one partially mouted on microscopic slide, Sumatra, Toba area, stream south of Balige (FT13), 8.IV.1929, Prof. Feuerborn leg [ZMH]; 2 nymphs, Sumatra, Toba area, Balige, stream at ca 1100 m (T13), 5.IV.1929, Prof. Feuerborn leg [ZMH] [All specimens *sub. nom* *Thalerosphyrus determinatus* det. Ulmer].

1 nymph, Sumatra Utara Province, swift stream 20 km East of Parlilitan (CL 2192), 1070 m, 10.XI.1985, J.T. & D.A. Polhemus leg [MZL]; 2 nymphs, Sumatra Barat, Tarusan, upstream Tarusan, 10 m, 100°29.84'E, 1°13.61'S, 24.V.2010 (SU3), J.-M. Elouard leg [MZL]; 2 nymphs, Sumatra Barat, Kotobarapak, upstream Kototbarapack, 100 m, 100°32.08'E, 1°13.78'S, 24.V.2010 (SU4), J.-M. Elouard leg [MZL]; 4 nymphs, Sumatra Barat, Lubukbargalung, Lubuk Paraku River, 50 km south Solok, 420 m, 100°32.50'E, 0°56.75'S, 25.V.2010 (SU5), J.-M. Elouard leg [MZL].

Eggs extracted from the mature female nymph mentioned above from Polhemus collected specimens.

#### Sequence data.

Three specimens (SU3, SU4, SU5) have been used for the study by Vuataz et al. (2013) under the name “*Thalerosphyrus*” in figures and “*Thalerosphyrus* sp.” in table S1, with voucher numbers “319SuTh”, “317SuTh” and “339SuTh” respectively, with one or two mitochondrial (CO1, 16S) and two to four nuclear genes (28S, H3, wg, EF-1α) sequenced. Access numbers in GenBank are:

**Table:** 

Voucher #	CO1	16S	28S	H3	wg	EF-1α
319 SuTh	HE651389					
317SuTh	HE651388	HE651425	HE651450	HE651508	HE651480	HE651532
339SuTh	HE651393	HE651429		HE651511	HE651484	HE651534

#### Description of the nymph.

Body size: up to 21 mm (full grown female nymph).

Coloration pattern: see Figs 7–8.

*Head.* Labrum greatly expended laterally, ca 4 times larger than long, with narrow and somewhat acute apexes (Fig. 17); dorsal surface and anterior margin covered with long and thin setae; ventral surface with a long median arch of ca. 20 strong and pointed setae ending close to the anterior margin. Crown of the galea-lacinia of the maxillae composed of ca. 20 comb-shape setae, the median ones bearing 12–14 teeth. Right mandible (Fig. 19) with 11–12 fimbriate setae below the inner incisor and 5 long simple and thin setae below the mola; left mandible (Fig. 18) with 8–9 fimbriate setae below the inner incisor and ca. 8–9 long simple and thin setae below the mola. Hypopharynx with robust lingua bearing a tuft of small setae, superlinguae densely covered with long and thin setae up to the lower part of the superlinguae (Fig. 28). Labium with glossae rhomboid, clearly concave on their inner and outer margins near apex (Fig. 22), dorsal surface with numerous stout setae and numerous thin and simple setae.

*Thorax.* Pronotum greatly expended laterally and posteriorly (Fig. 7). Femora with submarginal rows of pointed bristles on the inner and outer margins, only slightly increasing in numbers from the fore to the hind leg. Bristles on the upper face of hind femora with subparallel or slightly convergent margins, apex rounded or truncate (Fig. 33). Outer margin of hind tibia with a row of 12–15 pointed bristles in marginal or submarginal position (Fig. 34). Tarsal claw with 3–4 teeth.

*Abdomen.* Posterolateral expansions not developed on segments I–II, moderately developed on segment III and strongly increasing in size up to VIII where they may be longer than segment IX (Fig. 9). Gill I with asymmetrical elongated and rounded plate, less than two times longer than wide (Fig. 46); gill III–VI strongly asymmetrical, wider than long (Figs 47–48), gill VII oval and asymmetrical with slightly pointed apex (Fig. 49). Posterior margin of tergites with long and pointed teeth regularly alternating with two small ones, and few microdenticles (Fig. 37). Cerci whitish with 3–4 dark brown bands increasing in size towards the apex.

#### Description of the eggs.

Size: ca 140–150 µm × 85–90 µm; chorion regularly covered by pedunculate KCT'S, (1.0–1.5 µm), a little bit larger at poles (Fig. 14), no micro- or mesogranules present; margin of micropyle edged, as formed by fused peduncles (Fig. 15).

#### Discussion.

A major surprise was to find nymphs of *Thalerosphyrus lamuriensis* among the material identified by Ulmer (1939) as *Thalerosphyrus determinatus*, because he described this nymph based on a single specimen under the name *Ecdyonurus sumatranus* (Ulmer, 1939, see Sartori 2014d for a complete development of this case). *Thalerosphyrus lamuriensis* clearly differs from the two previous species by several characters, such as the posterolateral expansions of the abdomen reaching their largest size on segment VIII (compared to segment VII in *Thalerosphyrus determinatus* and *Thalerosphyrus sinuosus*), by the setation of the hypopharynx with long setae up to the concave margin of the superlinguae, the shape of the pronotum, the shape of the bristles on the upper face of hind femora, and the ornamentation of the hind tibiae. Together with *Thalerosphyrus vietnamensis* Dang, 1967, *Thalerosphyrus bishopi* Braasch & Soldán, 1986 and *Thalerosphyrus flowersi* Venkataraman & Sivamarakrishnan, 1987, *Thalerosphyrus lamuriensis* constitutes a group called by Kluge (2004) Ecdyonuroides/g(1) and characterized by “posterolateral projections […] on segments VI–VIII very long and pointed, exceeding segment length”. The three above mentioned species are incompletely described, but *Thalerosphyrus lamuriensis* differs from them apparently by the shape of the bristles on the upper face of femora, by the shape of the first gill and by the coloration of the abdomen (Braasch and Soldán 1984).

*Thalerosphyrus lamuriensis* possesses anyway far more characters in common with *Thalerosphyrus determinatus* and *Thalerosphyrus sinuosus* than the observed (although quite obvious) differences, and there is no reason on this basis to propose other generic rearrangement for Ecdyonuroides/g(1).

Eggs of *Thalerosphyrus lamuriensis* are very peculiar with pedunculate KCT'S, which distinguish them from the two other species.

*Thalerosphyrus lamuriensis* is the most abundant *Thalerosphyrus* species in Sumatra, and seems widespread throughout the island. In several places, it has been found together with *Thalerosphyrus sinuosus*.

### Key to the *Thalerosphyrus* nymphs occurring on Java and Sumatra

**Table:** 

1	Posterolateral expansions on the abdomen greatly enlarged (Fig. 8), reaching their maximum on segment VIII; protonum greatly enlarged laterally (Fig. 7); bristles on the dorsal face of hind femora truncate or rounded at apex (Fig. 33); hypopharynx with outer margin of superlinguae evenly covered with long setae (Fig. 28)	*Thalerosphyrus lamuriensis*
–	Posterolateral expansions of the abdomen more or less developed, those of segment VIII always shorter than those of segment VII (Figs 3, 6); pronotum moderately enlarged laterally; bristles on the dorsal face of hind femora arrow-shaped (Figs 29, 31); hypopharynx with outer margin of superlinguae covered with long setae ending at apex by minute setae (Fig. 27)	2
2	Hind tibia with only two rows of thin setae (Fig. 30); posterolateral expansions of the abdomen weakly developed (Fig. 3); gill I more than 2.5 times longer than wide (Fig. 38)	*Thalerosphyrus determinatus*
–	Hind tibia with two rows of thin setae and a submarginal row of arrow-shape bristles (Fig. 32); posterolateral expansions of the abdomen strongly developed (Fig. 6); gill I less than 2 times longer than wide (Fig. 42)	*Thalerosphyrus sinuosus*

### Notes on the male imaginal stages

The ZMH collections housed few male imagos of *Thalerosphyrus*, namely a single male of *Thalerosphyrus determinatus* and two of *Thalerosphyrus sinuosus*. These have been described in details by Ulmer (1913; 1924; 1939). Both species differ by the shape of the genitalia, by the coloration of the abdomen and by the tarsal composition of the hind legs. The report and description by Ulmer (1924) in Sumatra (Gunung Dempu, 1400 m, VIII.1916, Jacobson leg) of a male imago of the Philippine species “*Thalerosphyrus*” *torridus* (Walker, 1853) has already been considered as highly dubious by Braasch (2011). This specimen anyway displays general characteristics of the genus *Thalerosphyrus*, but differs from *Thalerosphyrus determinatus* and *Thalerosphyrus sinuosus* by the shape of the genitalia, and the tarsal composition of the hind leg. It is possible and even probable that this specimen represents the male stage of the species *Thalerosphyrus lamuriensis*, but only in situ rearing may bring the definitive proof.

## Supplementary Material

XML Treatment for Thalerosphyrus

XML Treatment for Thalerosphyrus determinatus

XML Treatment for Thalerosphyrus sinuosus

XML Treatment for Thalerosphyrus lamuriensis
